# Report from a symposium on catalyzing primary and secondary prevention of cancer in India

**DOI:** 10.1007/s10552-015-0637-x

**Published:** 2015-09-03

**Authors:** Suneeta Krishnan, Preet K. Dhillon, Afsan Bhadelia, Anna Schurmann, Partha Basu, Neerja Bhatla, Praveen Birur, Rajeev Colaco, Subhojit Dey, Surbhi Grover, Harmala Gupta, Rakesh Gupta, Vandana Gupta, Megan A. Lewis, Ravi Mehrotra, Ann McMikel, Arnab Mukherji, Navami Naik, Laura Nyblade, Sanghamitra Pati, M. Radhakrishna Pillai, Preetha Rajaraman, Chalurvarayaswamy Ramesh, G. K. Rath, Richard Reithinger, Rengaswamy Sankaranarayanan, Jerard Selvam, M. S. Shanmugam, Krithiga Shridhar, Maqsood Siddiqi, Linda Squiers, Sujha Subramanian, Sandra M. Travasso, Yogesh Verma, M. Vijayakumar, Bryan J. Weiner, K. Srinath Reddy, Felicia M. Knaul

**Affiliations:** Research Triangle Institute Global India Pvt. Ltd, Suite 405, Paharpur Business Center, 21 Nehru Place, New Delhi, 110019 India; Centre for Chronic Conditions and Injuries, Public Health Foundation of India, Sector 44, Gurgaon, 122002 India; Harvard Global Equity Initiative, Harvard University, 651 Huntington Avenue, Room 632, Boston, MA 02115 USA; Independent Public Health Consultant, 2C Alsa Terraces, 26 Langford Gardens, Bangalore, 560025 India; Chittaranjan National Cancer Institute, 37, S. P. Mukherjee Road, Kolkata, 700026 India; Department of Obstetrics & Gynaecology, All India Institute of Medical Sciences, Ansari Nagar, New Delhi 110029 India; Biocon Foundation, 20th KM Hosur Road, Electronic City, Bangalore, 560100 India; RTI International, 701 13th St NW #750, Washington, DC 20005 USA; Indian Institute of Public Health-Delhi, Public Health Foundation of India, Plot 47, Sector 44, Gurgaon, 122002 India; University of Pennsylvania, 3400 Civic Center Blvd, Philadelphia, PA 19104 USA; CanSupport, KanakDurgaBastiVikasKendra, Sector 12 R. K. Puram, Near New CGHS Dispensary, New Delhi, 110022 India; Rajasthan Cancer Foundation, B-113, 10 B Scheme, Gopalpura Bypass, Jaipur, 302018 India; V Care Foundation, A102, Om Residency, J W Road, Near Tata Memorial Hospital, Parel (East), Mumbai, 400012 India; RTI International, 3040 Cornwallis Rd., PO Box 12194, Research Triangle Park, NC 27709-2194 USA; Institute of Cytology and Preventive Oncology (ICMR), I-7, Sector-39, Noida, 201301 India; American Cancer Society, Inc., 250 Williams Street NW, Atlanta, GA 30303 USA; Center for Public Policy, IIM Bangalore, Bannerghatta Road, Bangalore, 560076 India; Indian Institute of Public Health Bhubaneswar, Public Health Foundation of India, Infocity Road, Patia, Bhubaneswar, 751024 India; Rajiv Gandhi Centre for Biotechnology (Government of India, Ministry for Science and Technology), Millennium Avenue, Jagathy, Thiruvananthapuram, 695014 India; Center for Global Health, National Cancer Institute, 9609 Medical Center Drive, Rockville, MD 20892-9760 USA; Kidwai Memorial Institute of Oncology, Dr. M.H Marigowda Road, Bangalore, 560029 India; All India Institute of Medical Science, Gautam Nagar, Ansari Nagar East, New Delhi, 110029 India; International Agency for Research on Cancer (WHO-IARC), 150 Cours Albert Thomas, 69372 Lyon Cedex 08, France; Tamil Nadu Health Systems Project, 3rd Floor, DMS Annex New Building 259 Anna Salai, Teynampet, Chennai, 600006 India; Cancer Foundation of India, 47/2D, Selimpur Road, Kolkata, 700031 India; RTI International, 1440 Main Street, Suite 310, Waltham, MA 02451-1623 USA; St. Johns Research Institute, 100 Feet Road, Koramangala, Bangalore, 560034 India; S.T.N.M Hospital, NH 31A, Gangtok, Sikkim 737101 India; Gillings School of Global Public Health, University of North Carolina at Chapel Hill, Chapel Hill, NC 27599-7411 USA; Public Health Foundation of India, Delhi NCR, Plot No. 47, Sector 44, Gurgaon, Haryana 122 003 India

**Keywords:** Cancer, Prevention, Policy, Advocacy, India, Symposium

## Abstract

**Purpose:**

Oral, breast, and cervical cancers are amenable to early detection and account for a third of India’s cancer burden. We convened a symposium of diverse stakeholders to identify gaps in evidence, policy, and advocacy for the primary and secondary prevention of these cancers and recommendations to accelerate these efforts.

**Methods:**

Indian and global experts from government, academia, private sector (health care, media), donor organizations, and civil society (including cancer survivors and patient advocates) presented and discussed challenges and solutions related to strategic communication and implementation of prevention, early detection, and treatment linkages.

**Results:**

Innovative approaches to implementing and scaling up primary and secondary prevention were discussed using examples from India and elsewhere in the world. Participants also reflected on existing global guidelines and national cancer prevention policies and experiences.

**Conclusions:**

Symposium participants proposed implementation-focused research, advocacy, and policy/program priorities to strengthen primary and secondary prevention efforts in India to address the burden of oral, breast, and cervical cancers and improve survival.

## Introduction

India bears over a tenth of the global burden of cancers [1]. Annually, approximately 1 million women and men are newly diagnosed with cancer and over 700,000 die as a result of their malignancies. Oral, breast, and cervical cancers account for a third of this burden despite the existence of feasible and cost-effective primary and secondary prevention methods [2]. With a view toward identifying necessary actions for effective implementation and scale-up of primary and secondary prevention strategies to reduce the burden of oral, breast, and cervical cancers in India, RTI International, Public Health Foundation of India (PHFI), American Cancer Society (ACS), Institute of Cytology and Preventive Oncology (ICPO) of the Indian Council of Medical Research, and Harvard Global Equity Initiative (HGEI) convened a symposium entitled, “Cancer Prevention in India: Catalyzing Action and Enhancing Implementation.” At this meeting, which was held in New Delhi on 19 and 20 February 2015, Indian and global experts deliberated on opportunities for and challenges related to primary and secondary prevention of cancers at national and state levels. Here, we present a summary of the discussions and recommendations for implementation-focused research, advocacy, and policies/programs to advance the primary and secondary cancer prevention agenda in India.

## Symposium objectives and structure

The objectives of the symposium were threefold. First, the meeting aimed to bring together a diverse group of stakeholders to discuss challenges and solutions related to strategic communication and implementation of cancer prevention, early detection, and treatment linkages. Stakeholders included those from government, academia, private sector (health care, media), donor organizations, and civil society (including cancer survivors and patient advocates). As specific health agendas and spending are primarily determined at the state level in India, representatives were invited from diverse states, particularly where large-scale cancer prevention activities are being planned or underway, including the national capital region, Karnataka, Kerala, Haryana, Maharashtra, Mizoram, Odisha, Rajasthan, Sikkim, Tamil Nadu, Telengana, Uttar Pradesh, and West Bengal. Experts from Bangladesh, Botswana, France, Sweden, Switzerland, and the USA also participated to share experiences and lessons. Second, participants highlighted innovative approaches to implementing and scaling up cancer prevention from India and elsewhere in the world. Third, participants were asked to reflect on existing global guidelines and national cancer prevention policies and experiences and to propose implementation-focused research, advocacy, and policy/program priorities to strengthen cancer prevention efforts in India.

Plenary talks focused on national and international perspectives on the primary and secondary prevention of oral, breast, and cervical cancers. Subsequent sessions focused on two themes—(1) strategic communication for cancer prevention and (2) prevention, early detection, and treatment linkages. Multiple stakeholders from different geographical and socioeconomic contexts (e.g., Indian states, other LMICs, high-income settings) offered their perspectives on the themes. Through breakout group discussions on key symposium thematic areas, participants selected the top priorities for research, advocacy, and policy/programmatic action to advance cancer prevention in India. The priorities were presented in a plenary session, and a panel comprising state and national government representatives as well as global stakeholders offered their reflections on the priorities in terms of importance and feasibility.

This symposium met an urgent need: Catalyzing primary and secondary cancer prevention efforts will require the inclusion and involvement of multiple stakeholders and stewardship to convene such partnerships. Effective implementation will require an educated and engaged community, knowledgeable and experienced health professionals, and the leadership of program implementers and policy makers—all working together. The conveners of the symposium—RTI, PHFI, ACS, ICPO, and HGEI—bring a diversity of local, national, and global experiences and resources related to cancer prevention and control as well as a commitment to work in partnership to reduce India’s cancer burden.

## Overview of presentations

### Background

The Government of India (GOI) has demonstrated a strong commitment to addressing non-communicable diseases (NCDs). In 2010, the National Programme for Prevention and Control of Cancer, Diabetes, Cardiovascular Diseases and Stroke (NPCDCS) was launched, with services being integrated under the National Health Mission [3]. The National NCD Monitoring Framework outlines 21 indicators and 10 targets, including a 25 % reduction in overall mortality from NCDs by 2025 [4]. Toward this end, GOI has announced the establishment of 20 state cancer institutes and 50 tertiary care centers with up to US$20 million and US$7 million assistance for each, respectively [5]. However, for these investments in treatment infrastructure to substantially reduce cancer-related morbidity and mortality, population-based prevention and early detection efforts through multi-stakeholder and multi-disciplinary approaches are needed.

### Challenges, opportunities, frameworks

The opening sessions of the symposium set the stage for discussions on the opportunities for and challenges related to multi-stakeholder, multi-disciplinary cancer prevention efforts in India. Speakers described the urgent need for primary and secondary cancer prevention activities given the growing burden of cancers and NCDs in India and other LMICs [6]. They noted that other LMICs, including India’s neighbors (e.g., Bangladesh, Bhutan, Malaysia, and Thailand), had developed national policies and engaged in local actions to address their cancer burden [7]. For example, Bhutan and Malaysia are implementing HPV vaccination as a part of their national immunization program. In Thailand, more than a million women have participated in the large-scale cervical cancer “screen-and-treat” program across 20 provinces. Speakers noted that there is a need for similar large-scale prevention efforts in India.

Several approaches were proposed to guide cancer prevention actions. It was noted that there are gross inequities in exposure to risk factors for cancers and the burden of preventable morbidity, mortality, and suffering from these diseases falls disproportionately on the poor [8]. As a means of overcoming this “cancer divide,” diagonal approaches—simultaneous focus on systemic gaps and disease-specific priorities across the life course and care continuum—to health systems strengthening were recommended [9, 10]. For example, explicit integration of cancer into universal health coverage reforms can offer an opportunity to strengthen health systems facing the challenge of chronicity [11], and the need to address palliative and end-of-life care needs [12]. The Mexican health reform exemplified this approach. In Mexico, cancer care was integrated into the *Seguro Popular*, the national health insurance program, and existing women and health programming was also harnessed to address women’s cancers [13]. Botswana’s longer-living HIV population who are increasingly diagnosed with cancer also highlights the need to address shortages in healthcare facilities, medical resources, and healthcare professionals that cut across diseases [14].

Social ecological frameworks were highlighted as a means to bridge the evidence-to-practice gap in cancer prevention by promoting an understanding of how individual, interpersonal, organizational, community, and macro-policy-level determinants interact to influence health and well-being [15]. Based on this understanding, interventions at different levels could be combined to produce complementary effects by capitalizing on causal inter-dependence between levels. Useful strategies for combining interventions at multiple levels included accumulation, amplification, facilitation, cascade, and convergence [15].

A major challenge facing cancer prevention efforts in India is the inadequacy of resource allocations for and expenditures on health [16]. Speakers underscored the need for increasing allocations and spending as well as the role of partnerships for achieving impact and sustainability. The triad of strong political will, careful financing, and strategic communication were highlighted as the key to the successes of cancer prevention programming in countries like Malaysia and Rwanda [11]. The Tamil Nadu Health Systems Project’s cancer prevention initiative (2007–2010) is a prime Indian example of the progress that can be made when cancer prevention has political backing and administrative leadership [17].

### Prevention, early detection and linkages to treatment, and palliative care

These sessions focused on challenges and opportunities for cancer prevention, early detection, and linkages to treatment. Implementation science was used as an organizing framework for these sessions because it enables the identification of implementation strategies that can address context-specific challenges, provide implementation support (e.g., through implementation tool kits), facilitate staging or phasing in implementation strategies by prioritizing what needs to be done first, and strengthen learning/improvement capacity of individuals, organizations, and systems [18].

Speakers noted that there are a range of oral, cervical, and breast cancer screening and early detection methods available for use in India. However, evidence on feasibility of implementation at scale, impact, and cost-effectiveness of these methods in India remained limited because there had been few attempts to implement these strategies outside of randomized trial settings. For example, the WHO in its latest guidelines on cervical cancer screening has recommended a single-visit “screen and treat approach,” in which women who screen positive (using visual inspection or HPV tests) are treated without any further diagnostic verification [19]. This has the potential to reduce non-compliance and improve program efficiency [20]. Programmatic experiences from Bangladesh brought forth the challenges in implementing a visual inspection-based screen-and-treat approach, including limited acceptability of screening, variability in the quality of service provision, and difficulties in data management for monitoring and evaluation. These experiences highlighted the need to examine the feasibility and potential impact of screen-and-treat strategies in India.

Cost-effectiveness of cancer screening and early detection was emphasized as an area that requires greater research attention [21]. Speakers noted that it is important to utilize activity-based costs and detailed quality indictors to evaluate both screening trials and demonstration projects to ensure that large-scale implementation efforts are designed and optimally resourced to achieve targeted program effectiveness and outcomes [22, 23]. It was noted that assessments of total cost of cancer screening can be misleading because resources expended on specific program activities can have direct impact across multiple dimensions including access, quality, and adherence to care, and these in turn can impact both overall healthcare cost and program effectiveness.

Pathways linking screening, early detection, treatment, and palliative care were also explored. Research in Odisha quantified the delays in care-seeking for signs and symptoms related to cancer [24]. The study found that the first step in the pathway-to-care was sharing symptoms or signs with family members and friends, and on average, took 271 days before steps toward diagnosis were taken. Lack of knowledge, fear, and stigma related to cancer were highlighted as the key factors influencing this delay. Symposium speakers also noted the importance of strengthening healthcare systems and improving quality of care in order to encourage timely care-seeking and follow-up. Adoption of a Charter of Rights of People Living with Cancer akin to the charter proposed for diabetes [25] and improvements in access to and quality of palliative care were cited as important steps [26–28].

Addressing the need for financial protection was underscored as critical to improving access to and utilization of cancer prevention and control services [11, 29]. A study of nearly 200,000 households across India using data from 1995 to 1996 and 2004 found that a single hospital stay for cancer accounted for 80–90 % of per capita income (INR 25,320 in 2004) if health care was obtained from a private provider compared to 40–50 % at a public facility. The odds of incurring catastrophic hospitalization expenditures were nearly 160 % higher with cancer compared to the odds of incurring catastrophic spending when hospitalization was due to a communicable condition [30]. Potential solutions to this challenge have been developed in a number of Indian states. Financial support for cancer treatment for households below the poverty line is being implemented through the Chief Minister’s Insurance Scheme in Tamil Nadu and the Vajpayee Arogyashree Scheme (VAS) scheme in Karnataka [29].

The sessions also entailed an examination of state-level experiences and perspectives on cancer prevention, early detection, and care linkages. The state of Tamil Nadu in southern India has integrated cervical and breast cancer screening into the existing healthcare system, including at the primary healthcare level, as a part of an NCD prevention and control program. Facilitators of state-level scale-up included: (1) mobilization of existing human resources within the public health system and longstanding women’s self-help groups to promote NCD screening, and (2) community outreach to men with messaging along the lines of “I care for my wife and I will take her to the screening center.” Challenges included those related to human resources (recruitment, attrition, and capacity building), infrastructure (clinic space, ensuring privacy), protocol adherence (deviations/incorrect practices, staff resistance to take up new procedures), social acceptability (motivating women to go for screening), finances (sustainability of program), logistics (difficulty in large-scale procurement of drugs and reagents), and health systems integration (coordination of follow-up and referrals). A number of strategies have been devised to overcome these challenges. For example, problems related to attrition of NCD staff nurses was overcome by outsourcing to a human resources agency. District officials were enlisted to help create private spaces within clinics to offer NCD-related services, and women’s self-help groups were mobilized to motivate women for screening. Sustainability of the program is being considered through continued support from the National Health Mission (NPCDCS).

Sikkim, a northeastern Indian state, has taken a camp-based approach to promoting oral, cervical, and breast cancer screening and treatment. In this model, teams comprising doctors, nurses, and paramedical workers offer screening at the village level on a selected day. Individuals who require further evaluation are referred to a tertiary hospital. State authorities are planning to scale up these efforts. Sikkim now plans to emphasize routine screening of breast, cervical, and oral cancer, timely referral of confirmed cases to empanelled hospitals, and primary prevention of cervical cancer through HPV vaccination.

The sessions concluded with a discussion of technological innovations to address the challenges associated with delivering prevention, early detection, and treatment linkages. Biocon Foundation has developed the Mobile Early Detection and Prevention of Oral Cancer (Medpoc) platform in which community health workers use a mobile phone application not only to screen for oral lesions, but also to identify high-risk individuals and to target counselling and follow-up [31]. The platform is being implemented in rural and urban communities in Karnataka, and opportunities for scale-up are being explored. Speakers also addressed the discovery and validation of biomarkers to facilitate population prediction and clinical management of cancers. The identification of biomarkers to facilitate the clinical management of oral cancer including staging and pathological classification of tumors and in cancer chemoprevention trials was discussed.

### Strategic communication for cancer prevention

Research in India has shown that lack of information and awareness about oral, breast, and cervical cancers is a critical barrier to timely detection and treatment and leads to poor outcomes [21]. Tobacco control efforts in India have been notable in this regard, particularly as a result of multi-stakeholder engagement and partnerships [32]. Strategic communication through collaborations between clinicians, public health promoters, cancer survivors, and journalists is critical to ensure dissemination of accurate information to the population at large [33]. Positive stories of cancer survivorship are likely to help change common negative perceptions of cancer such as the view that the disease is a death sentence. Speakers noted that negative stereotypes in the popular media exacerbated fear of the disease and stigma and were likely contributors to delays in care-seeking. Stigma is a harmful social process that undermines prevention, care, and treatment through labeling, associating negative attributes, social separation, and status loss and discrimination [34]. Research on HIV and other stigmatized illnesses demonstrates that communication can inadvertently cause stigma. That said, stigma can also be mitigated by raising awareness, discussing and challenging shame and blame, and addressing transmission fears and misconceptions [35].

Experts noted that it was essential to integrate strategic communication across the cancer care continuum and that such efforts should be framed keeping in mind the risk of creating stigma [36]. Strategic communication efforts are needed to promote cancer literacy (e.g., awareness of risks and prevention strategies), enhance social support for those affected by cancer, increase the accountability of health systems, and empower the public to demand cancer prevention and control services.

Several communication approaches were described. Mass media campaigns can be effective in changing people’s behaviors and impacting policies when guided by evidence. For example, the Alliance for Healthy Food (Mexico) implemented a mass media campaign in 2013 to raise knowledge about sugary drinks and their link to chronic diseases, which has resulted in a substantial increase in parental intentions to reduce their children’s intake of sugar-sweetened beverages [37]. Other important channels of communication that should be considered include interpersonal communication between healthcare providers and patients and dissemination of information through social networks. Speakers emphasized the need to evaluate strategic communication initiatives using these channels and focused on different population subgroups in India.

The Tamil Nadu government’s strategic communication for cancer prevention included the use of mass media (television and radio), print materials (posters, stickers, flipbooks, and pamphlets), and street plays, at a cost of approximately USD 3 million. Program experiences suggest that the television commercials had the broadest reach and impact.

Strategic communication strategies used in the USA were also shared. India may consider developing large-scale surveys similar to the US Health Information National Trends Survey, which is used to track knowledge, attitudes, and cancer-related behaviors of Americans [38]. A surveillance program in India could be used to plan and evaluate state, regional, or national prevention programs such as those focused on tobacco control, cancer screening, physical activity, nutrition, and cancer stigma. Cancer Control PLANET is an Internet-based platform used by public health professionals and national- and state-level policy makers to identify evidence-based cancer control programs [39]. PLANET includes state cancer control profiles that provide public health professionals with state-level data such as demographic characteristics, screening behaviors, cancer risk factors, cancer knowledge, and cancer incidence, prevalence and mortality. Recommendations and guidelines from key organizations such as the US Preventive Services Task Force and the Guide to Community Preventive Services are also included on the PLANET platform. PLANET has been available in the USA for over a decade and could be used as a model to create a platform for public health professionals in India.

These sessions resulted in the recommendations for implementation-focused research, advocacy, and policies/programs for improving initiatives focused on the two symposium themes.

## Research recommendations

At the end of 2 days, stakeholders suggested the following research priorities to advance the primary and secondary cancer prevention agenda in India.

### Prevention, early detection and linkages to treatment, and palliative care

- *Examine feasibility, acceptability*, *and impact of prevention and early detection strategies.* Participants recommended that approaches that have been tested in research settings should also be examined in program settings for feasibility, acceptability, and impact [40]. In the case of cervical cancer, these include “screen and treat” approaches, use of self-collected samples for HPV-based screening as well as use of VIA to triage HPV-positive women for treatment. Additional research on HPV vaccination is also needed, such as the efficacy of two-dose versus three-dose HPV vaccine regimens, and efficacy among adolescent girls living with HIV. Development of the next generation of vaccines with broader protection and affordable pricing was also encouraged [41].
- *Identify appropriate target populations for cancer prevention and control efforts.* Research is needed to determine the optimal age and risk-stratified groups to target oral, breast, and cervical cancers prevention efforts, building on evidence from trials and national recommendations [3, 22, 42–44]. Factors underlying the younger age at diagnosis for cancers such as breast cancer in India should be examined.
- *Estimate cost*-*effectiveness of prevention and early detection approaches.* Guidelines for the implementation of programs to prevent breast, cervical, and oral cancers are available from the WHO, international alliances, the GOI, and other sources (see, for example, [3]). However, participants concurred that when guidelines are operationalized as programs, data on cost-effectiveness should also be collected as an integral component of monitoring and evaluation as these data can be used to inform policy and program planning.
- *Identify effective, scalable methods to optimize health workforce and enhance health worker performance.* Optimal tasking—the merging of task shifting and task sharing mechanisms as appropriate in the context—is necessary to manage chronic care needs such as those for cancer. Strategies for optimal task shifting in the Indian context should be identified and evaluated [45]. In addition, programs to enhance and sustain health workers’ motivation and skills to deliver high-quality cancer prevention services should be developed and tested. Engagement of practitioners of Ayurveda, Unani, Siddha and Homeopathy (AYUSH), community volunteers, and medical/nursing/dental colleges should also be explored.
- *Identify strategies to improve follow*-*up and care linkages.* To enable the establishment of efficient referral and follow-up systems to improve patient care, implementation science research is needed to understand the barriers to and facilitators of screening, early detection, follow-up, treatment initiation and completion, and their impacts on survivorship. Research should implement and evaluate strategies that address these factors and effectively improve care linkages. Studies may include those focused on the design of and incentives for screening and diagnosis, including service delivery in rural, remote communities. Research is also needed to identify how promising approaches can be scaled up and sustained.
- *Examine technology innovations in the continuum of care.* The role of technology including point-of-care tests for screening and diagnosis, mobile phone technology, and telemedicine in strengthening care linkages, should be examined. Evaluations of technological innovations should assess multiple dimensions of performance, such as provider practices, care linkages, healthcare costs, and patients’ quality of life [46].
- *Adapt evidence*-*based guidelines to different settings.* A major area of study is the application of cancer prevention guidelines across Indian states with different cultural contexts and resource levels. Lessons can be learned from the implementation experiences of India’s neighbors such as Bangladesh (in cervical cancer screening) [7], Sri Lanka (for oral cancer screening) [47, 48], and Bhutan and Malaysia (in HPV vaccination) [7].
- *Identify barriers to pain control and palliative care and develop effective strategies to increase access.* Participants noted that despite policy-level progress on pain control and palliative care in India, barriers to providers prescribing and patients using pain medication remain [49]. More qualitative and quantitative research is needed to better understand the barriers to pain control and palliative care and to facilitate the translation of policies into routine practice.

### Strategic communication

- *Identify effective approaches to improve cancer literacy.* Cancer literacy, which may be defined as enhancing individuals’ access to and understanding of cancer and cancer prevention and control to support informed health decision-making [50], must be improved across all segments of India’s population and subgroups, including men and women, healthcare providers, policy makers, patients, families and care givers, and health promotion organizations. Research is needed to better understand the information needs of these population groups as well as how information is shared and transmitted within populations. Research is also needed to identify the most effective cancer prevention messages and channels for message dissemination. For example, studies are needed to assess the role of social media platforms [51–53] as well as the potential for leveraging mobile phone technologies for improving access to health information [54]. Message testing should be conducted prior to broader dissemination to ensure that messages will resonate with the target audiences.
- *Understand social and cultural barriers to cancer prevention.* Research should focus on understanding and addressing the role of a range of factors such as stigma and discrimination, fear, fatalism, predetermination, and gender inequity as barriers to cancer screening, early detection, and linkages to care [35, 36, 55, 56]. Such data can inform strategic communication initiatives across the care continuum.
- *Understand the drivers of behavior change.* Research is needed to identify the drivers of cancer prevention-related behaviors. Theory-informed studies on factors that influence cancer risk factors such as smoking, diet, and exercise as well as those that drive screening and treatment-seeking behaviors can provide the foundation for future efforts to promote positive behavior change.
- *Examine the role and cost*-*effectiveness of different communication channels.* A variety of communication channels such as mass media campaigns and interventions to improve interpersonal communication between providers and patients are available for cancer prevention. However, in the context of limited resources, the costs and effectiveness of these channels for improving cancer prevention outcomes become critical factors to consider in program planning [33].

## Recommendations for advocacy efforts

### Prevention, early detection, and linkages to treatment and palliative care

- *Include cancer care (early detection, treatment*, *and palliative care) as part of the essential packageof care*. Advocacy efforts for universal health coverage should include access to cancer prevention and control as part of services included under universal health coverage. Cancer treatment drugs as well as diagnostics and outpatient procedures should be included. Practices by health insurance companies such as exclusion of cancer survivors by treating cancer as a preexisting condition must be eliminated, and treatment should be made more affordable [57, 58].
- *Increase access to palliative care:* Hospital-based and home-based care models should be provided, as appropriate [26]. Access to palliative care is imperative to reduce suffering across the care continuum and particularly given that the majority of cancer patients in India are diagnosed at advanced stages of disease.
- *Increase multi*-*stakeholder collaboration.* Collaborations between public, private, and nonprofit players including different government ministries to address cancer prevention and control initiatives should be formed and learn from efforts in tobacco control in India [32].
- *Establish platforms for information exchange and dissemination.* A public health analogue to the treatment-focused National Cancer Grid can facilitate exchanges and partnerships among stakeholders in cancer prevention and control [59]. Such a platform can help disseminate lessons learned on a range of issues related to primary and secondary prevention of cancers, promote regional collaborations (e.g., southern states), and influence policy making.

### Strategic communication

- *Communicate the role of different systems of medicine in cancer care.* Advocacy efforts can help leverage India’s medical pluralism to advance cancer prevention. Although research on the engagement of AYUSH in cancer prevention is needed, advocacy efforts can support the investment of resources to generate and apply such information.
- *Encourage community volunteers as advocates.* Overall, advocacy groups (typically, cancer societies, survivors, and other volunteers) are relatively few in number in LMICs, including India [60]. In India, cancer survivors should be engaged to help disseminate prevention messages. Their involvement may not only help reduce fear and stigma related to cancer but also help address human resource shortages in prevention programs, and thus increase programmatic capacity to serve patients and their families. Community-based women’s self-help groups are another important community stakeholder that can be engaged as in Tamil Nadu.
- *Develop communication tools to support mobilization and advocacy.* Skills and capacities need to be developed to ensure that cancers remain a priority of NCD prevention and control programs and are adequately addressed in national policies. Communication tools and resources should be developed and deployed to this end.
- *Empower people living with cancer.* Tackling gender inequity, fear, and stigma and discrimination fear requires patient empowerment. People living with cancer should be aware of their rights (such as those under the Declaration of the Rights of People with Cancer [61]) and empowered to make decisions regarding the best course of treatment (e.g., in choosing between treatment options or terminating treatment at end-of-life stages). Patients should be supported by navigators, ideally at the community level, to access efficient pathways to diagnosis and treatment.
- *Sensitize media.* Symposium participants noted that myths and misconceptions about cancer, which are spreading quickly as a result of the Internet, mobile technology and social media, should be tackled through plain language and counter-messaging that present both facts and personal stories. Media should be encouraged and supported to be more sensitive in their coverage of cancer and in changing the image of “cancer” from one of death to one of life and survival.

## Policy/program recommendations

Symposium participants identified the following crosscutting policy and program recommendations:

- *Address the specificities of cancers in NPCDCS.* India’s national cancer control program, which has been in place since 1975, was integrated into the NPCDCS along with other NCDs in 2010. Cancer is a heterogeneous set of conditions with some risk factors (e.g., tobacco, alcohol, overweight/obesity, and physical inactivity) common to other NCDs. However, in addressing cancer, India’s NPCDCS and national health policy must also consider the unique dimensions of cancers, such as the high cost of diagnosis and treatment, infectious and environmental causes, and high levels of fear and stigma.
- *Increase quantum and efficiency of public health expenditure on health and cancer prevention.* Participants unanimously called for an increase in public health spending to 2.5 % of GDP from the current levels, which are among the lowest in the world (<1 %), and for increased spending on cancer prevention [57]. Funds need to be allocated to states and within states based on the state-level cancer scenario. Cancer and other NCD patients are at much greater risk of catastrophic health spending than those affected by communicable diseases [30]. Moreover, there is a need for better regulation of prescription practices and unfairly priced drugs, which constitute the majority of costs for cancer patients. Access to affordable palliative treatment is required [28], and states should ensure free, or at a minimum, reimbursable pain control and palliative care.
- *Streamline administrative processes.* Participants noted that unspent funds as a result of administrative and bureaucratic indecision at the national and state levels posed significant hurdles to initiation and/or implementation of cancer prevention and control programs. Delayed release of funds from the national government to the states and under-spending at the state level result in reduced budgets in successive years. Moves should be made to ensure the release of funds in the first quarter of the fiscal year in order to facilitate spending and program implementation. The Tamil Nadu Health Systems Project reduced administrative barriers through the issuance of government orders and streamlining of bureaucratic procedures and can serve as a model for other states.
- *Establish robust information systems.* Information systems such as electronic data capture, registries and surveillance of cancer-related knowledge, attitudes, beliefs, and behaviors of both the public and healthcare professionals are needed to better identify priority intervention areas. Registry and surveillance data can also be used to plan and evaluate programs [62].
- *Utilize a stepwise approach to screening and early detection programs.* A stepwise approach entails introduction of screening and early detection using the most acceptable and feasible test (such as VIA for cervical cancer) with the introduction of new strategies and technologies (such as HPV testing) as evidence accumulates and resources become available [63].
- *Strengthen different levels of the healthcare system.* Participants emphasized the importance of strengthening the role of primary health care in cancer prevention and establishing a stepped-care system [64]. Primary health care should be strengthened to raise awareness, assess risks, offer risk reduction interventions, and implement screening and referrals. District-level or secondary hospitals can offer diagnosis, certain types of treatment and palliative care, and facilitate referrals, while tertiary care centers can focus on provision of treatment as well as monitoring and evaluation of the geographic area under their coverage. Mechanisms to ensure accountability and coordination between these levels of the healthcare system also need to be established.
- *Increase human resources for cancer prevention.* There is an urgent need to address the shortage of human resources for cancer prevention. State and national governments should utilize existing cancer prevention planning tools to estimate human resource needs [65]. Mobilizing public and private medical, nursing, and dental colleges to integrate cancer prevention into their curricula and train and deploy staff and students is one approach to addressing these needs [66]. In-service education should be made available across states. Participants noted that the acceptability of screening and early detection initiatives focused on women may be enhanced by the availability of trained female health workers.
- *Invest in strategic communication efforts*, *including mass media campaigns.* Strategies should be responsive to the local cultural context. For example, given the stigma around sexually transmitted infections, campaigns should be careful about how the links between HPV and cervical cancer are communicated. Campaigns should focus on reducing stigma, emphasize cancer survivorship, and change the public perception about cancer as a death sentence.
- *Promote awareness and use of the HPV vaccine.* HPV vaccine initiatives should adapt successful promotional efforts implemented by regional neighbors such as Bhutan and Malaysia [57] and LMICs such as Rwanda, South Africa [67], Brazil, and Peru [68, 69]. Symposium participants noted that evidence from India and other LMICs had demonstrated the safety of the HPV vaccines, and information about vaccine safety and efficacy should be widely disseminated. Furthermore, the national immunization program should include HPV vaccination.
- *Improve inter*-*sectoral coordination.* There is a need to engage non-health sectors in cancer prevention and control efforts, similar to the work that has been done on tobacco control, which has included civil society organizations, private sector, and government ministries such as the Ministry of Information and Broadcasting, Ministry of Finance, and Ministry of Human Resource Development. Moreover, cancer and NCD prevention efforts can work synergistically with efforts to improve the health status of adolescent girls, children, and pregnant women.

A summary of the above recommendations was submitted in response to the GOI’s draft National Health Policy, which was available for public comment at the time of the symposium.

## Conclusion

Overall, several gaps in evidence, and challenges to implementation were noted in delivering effective communication on cancer prevention, and in ensuring better early detection and linkages to treatment across different Indian states and LMIC settings. That said, lessons and successes from cancer and other health outcome experiences for primary and secondary prevention, as well as innovations in health systems and technologies to improve treatment linkages, were discussed and recommended for practical, cost-effective steps forward. The importance of engaging multiple stakeholders across society and different disciplines, states, and countries was highlighted for successful, concerted collaborations, including the development of a task force to build on these efforts and monitor the progress and activities outlined in these recommendations in the future.

## Appendix

**Table:** Symposium agenda

Thursday, 19 February 2015	
9:30–10:00	**Session 1**: **Welcome** Welcome; RTI International and Global NCD Prevention *Wayne Holden*, *RTI International* Overview of India’s cancer challenge and symposium objectives *Preet Dhillon*, *PHFI, and Suneeta Krishnan*, *RTI International*
10:00–10:45	**Session 2**: **National and global perspectives on cancer prevention** Chairperson: *Ravi Mehrotra*, *ICPO* Indian healthcare system and the challenge of NCD prevention *K. Srinath Reddy*, *PHFI* Mobilizing local action for global change *Sally Cowal*, *ACS* Cancer prevention and the equity imperative *Felicia Knaul*, *HGEI*
10:45–11:00	Discussion
11:15–12:00	**Session 3: Cancer prevention frameworks and approaches** Chairperson: *Neerja Bhatla*, *AIIMS* A Framework for Bridging the Evidence to Practice Gap in Cancer Prevention *Megan Lewis*, *RTI International* Cancer prevention in India and other LMICs: what has worked and what are the challenges? *R. Sankaranarayanan*, *IARC/WHO*
12:00–12:30	Discussion
12:30–13:30	*Lunch*
13:30–14:30	**Session 4**: **Health systems perspectives on cancer prevention** Chairperson: *Richard Cash*, *Harvard/PHFI* and *Afsan Bhadelia*, *HGEI* Indian health system: framework and resources *V. R. Raman*, *PHFI* Generating rigorous data from India on what works: research on cervical cancer prevention and early detection *Partha Basu*, *CNCI*
	Cervical cancer screening in Bangladesh *Ashrafunnessa*, *BSMMU* Clinical trial to implementation: cost-effectiveness considerations for scaling up cancer screening *Sujha Subramanian*, *RTI* *International*
14:30–15:00	Discussion
Thursday, 19 February 2015 (continued)	
15:00–16:00	**Session 5: Implementing screening, early detection, and financial protection: state-level experiences** Chairpersons: *R. Sankaranarayanan*, *IARC/WHO* Using implementation science to advance cancer prevention *Bryan J. Weiner*, *University of North Carolina* PANEL: Scaling up cervical and breast cancer screening, early detection, and treatment linkages in Tamil Nadu *M. S. Shanmugam*, *TNHSP*, *Government of Tamil Nadu* Perspectives on cervical cancer prevention in Sikkim *Kumar Bhandari*, *Government of Sikkim* Karnataka’s experience with cancer care for people below the poverty line through Vajpayee Arogyashree *Arnab Mukherji*, *IIM Bangalore*
16:00–16:45	Discussion, wrap-up on key implementation challenges faced at the state level
16:45–17:00	Tea/Coffee
17:00–17:30	**Session 6: Keynote address** Chairperson: *G. K. Rath*, *AIIMS* Cancer prevention in India: the NPCDCS experience *C. K. Mishra*, *MOHFW*
17:30–18:00	**Session 7: reflections on government initiatives on cancer prevention in India** PANEL: MOHFW, Indian Council of Medical Research, Department of Science and Technology, Department of Biotechnology
18:00–18:30	**Innovation feature** Presentation: why biomarker discovery matters *Susan Sumner*, *RTI International* *Discussant*: *M. Radhakrishna Pillai*, *Rajiv Gandhi Centre for Biotechnology*
Friday, 20 February 2015	
9:15–9:30	**Registration**—**Welcome** *Tim Gabel*, *RTI International*
9:30–10:20	**Session 8: Multistakeholder perspectives on pathways to care** Chairpersons: *Subhojit Dey*, *PHFI* PANEL: Insights from research on pathways to cancer care *Sanghamitra Pati*, *IIPH Bhubaneswar*, *PHFI* Patient perceptions *Jyotsna Govil*, *ICS* Challenges in follow-up in community-based oral cancer screening *Praveen Birur N*., *Biocon Foundation* Accessing pain relief and palliative care in India *Harmala Gupta*, *CanSupport*
10:20–10:50	Discussion
10:50–11:00	Coffee/tea break
11:00–11:40	**Session 9: Innovations in pathways to care**—**global lessons** Chairperson: *Kanchan Kaur*, *Medanta* PANEL: Cancer care in Botswana: challenges and future directions *Surbhi Grover*, *University of Pennsylvania* Using mHealth to support disease surveillance and care linkages in global settings *Rajeev Colaco*, *RTI International* Closing the cancer divide: a diagonal approach to health systems strengthening *Afsan Bhadelia*, *HGEI*
11:40–12:00	Discussion, wrap-up on pathways to care: challenges and opportunities
Friday, 20 February 2015 (continued)	
12:00–12:45	**Session 10**: **multisectoral and multistakeholder perspectives on strategic communication** Chairperson: *Rakesh Gupta*, *Rajasthan Cancer Foundation* PANEL: Tobacco control to cancer control in India: heath promotion approach *Monika Arora*, *PHFI* Media perspective *Malathy Iyer*, *TOI Mumbai* Civil society efforts *Sutapa Biswas*, *Cancer Foundation of India* Survivors’ perspective *Vandana Gupta*, *V Care Foundation*
12:45–13:15	Discussion
13:15–14:15	Lunch, communications marketplace, and networking
14:15–14:45	**Session 11**: **Strategic communication for prevention**—**global lessons** Chairperson: *Navami Naik*, *ACS* Lessons from cancer communication efforts in the United States *Linda Squiers*, *RTI International* Insights from addressing HIV stigma globally and in India for Cancer stigma research and interventions *Laura Nyblade*, *RTI International* Mass media campaigns for health promotion: experiences from LMICs *Nandita Murukutla*, *World Lung Foundation*
14:45–15:15	Discussion, wrap-up on strategic communication: challenges and opportunities
15:15–15:30	**Session 12**: **Developing a policy and implementation-focused research agenda** Overview of group exercise and assignment
13:30–16:30	**Session 13**: **Group work** Two groups refining policy and research agenda focused on Overcoming obstacles in delivering prevention, early detection, and treatment linkages Advancing strategic communication for prevention
Friday, 20 February 2015 (continued)	
16:30–17:00	**Session 14**: **Plenary and discussion of recommendations** Chairpersons: *Maqsood Siddiqi*, *CFI*; *Doris Rouse*, *RTI International* 5-min presentation from groups, followed by discussion
17:00–17:30	**Session 15: Perspectives on funding cancer prevention research in India** Chairperson: *Preetha Rajaraman*, *NCI* PANEL: *Ann McMikel*, *ACS*, *R Sankaranaryanan*, *IARC*, *Yogesh Verma*, *Sikkim*, *Eric Zomawia*, *Mizoram*, *Jerard Selvam*, *Tamil Nadu*
17:30–17:45	Vote of thanks
